# The Law of Recency: An Episodic Stimulus-Response Retrieval Account of Habit Acquisition

**DOI:** 10.3389/fpsyg.2019.02927

**Published:** 2020-01-15

**Authors:** Carina G. Giesen, James R. Schmidt, Klaus Rothermund

**Affiliations:** ^1^Department of Psychology, Friedrich Schiller University Jena, Jena, Germany; ^2^Department of Psychology, Université Bourgogne Franche-Comté, Dijon, France

**Keywords:** law of recency, law of exercise, law of effect, habit acquisition, stimulus-response binding, event files, episodic response retrieval, contingency learning

## Abstract

A habit is a regularity in automatic responding to a specific situation. Classical learning psychology explains the emergence of habits by an extended learning history during which the response becomes associated to the situation (learning of stimulus-response associations) as a function of practice (“law of exercise”) and/or reinforcement (“law of effect”). In this paper, we propose the “law of recency” as another route to habit acquisition that draws on episodic memory models of automatic response regulation. According to this account, habitual responding results from (a) storing stimulus-response episodes in memory, and (b) retrieving these episodes when encountering the stimulus again. This leads to a reactivation of the response that was bound to the stimulus (c) even in the absence of extended practice and reinforcement. As a measure of habit formation, we used a modified color-word contingency learning (CL) paradigm, in which irrelevant stimulus features (i.e., word meaning) were predictive of the to-be-executed color categorization response. The paradigm we developed allowed us to assess effects of global CL and of an instance-based episodic response retrieval simultaneously within the same experiment. Two experiments revealed robust CL as well as episodic response retrieval effects. Importantly, these effects were not independent: Controlling for response retrieval effects eliminated effects of CL, which supports the claim that habit formation can be mediated by episodic retrieval processes, and that short-term binding effects are not fundamentally separate from long-term learning processes. Our findings have theoretical and practical implications regarding (a) models of long-term learning, and (b) the emergence and change of habitual responding.

## Introduction

In the cafeteria, you might notice that you bought some fries for lunch – yet again – instead of the much healthier salad. After a long day at work, you might find yourself taking the way home to your old place rather than the new one you recently moved to. Everyone knows situations like these, in which we behave by mere force of habit, sometimes even against our good intentions. But how did we acquire these habits? What is the source of habitual behavior? Psychologists have pondered over the processes underlying habit formation for over a century now.

Currently, the theoretical terrain on habit acquisition is dominated by two accounts, based on either the “law of effect” or the “law of exercise” (for overviews, see, e.g., Wood and Rünger, 2016; Wood, 2017; Miller et al., 2019). Early accounts explained habit acquisition in terms of operant conditioning (Thorndike, 1898; Hull, 1943). According to Hull (1943), habit strength is a direct function of the reinforcement history of a particular response in a specific situation. Whereas responding is initially based on the trial-and-error principle, the likelihood of showing a particular response again in a given situation will increase if the response was rewarded, but will decrease if the response was punished in the past. This emergence of habits for behaviors that were reinforced before is called the “law of effect” (Thorndike, 1898). Learning psychology has seen some debates of what counts a reward or reinforcer, with suggestions ranging from stimuli that reduce states of deprivation of biological needs and that are adaptive for survival (Hull, 1943), to more formal definitions focusing on the transituationally stable quality of a stimulus to increase the probability of different behaviors of a specific organism (Meehl, 1950), to opportunities to execute behaviors that are chosen with high frequency under free-choice conditions (Premack, 1965). A detailed discussion of these accounts is beyond the scope of this article, but it is evident that rewards can also be subtle effects and qualities of the behaviors that are studied. We will take up this important point again in the General Discussion (section “What Is a Reward?”).

Even early learning psychology, however, already had another explanation of habit acquisition that was independent of reinforcement: According to the “law of exercise,” habits can emerge as a mere result of repeating the same behavior in the same situation over and over again (Thorndike, 1898). Since reinforcement and repetition are typically confounded, the outcome devaluation paradigm has been used in order to assess habitual behavior that is independent of reward or valuable outcomes (Dickinson, 1985). Several studies have shown that although outcomes have a strong influence on instrumental behavior, behavior that has been highly overlearned in many repetitions continues to be shown even in the absence of reward or after the outcome has lost all its reinforcing qualities. For instance, the behavior might still be present after having paired the outcome with shock or after providing so much of the reward (e.g., food) that the animal is completely satiated, resulting in a refusal to consume the previously rewarding outcome when it is available (e.g., Rescorla, 1991; Colwill, 1993). These findings provide unambiguous evidence that mere repetition of a response can produce habitual behavior independently of expected reward or reinforcement. In sum, then, the concept of a habit captures the fact that behaviors eventually are elicited in a more or less automatic fashion by situational cues, even in the absence of rewards and intentions.

The concept of a habit can be broadly defined to reflect automatic operant behavior that is elicited by certain stimuli or situations. According to this definition, habitual behavior is necessarily characterized as being automatic, although the reverse does not hold: Behaviors can share features of automaticity, without necessarily reflecting habitual behavior (e.g., Amodio and Ratner, 2011). For instance, behavior that is based on instincts or autonomous reflexes (“respondent behavior”) can operate automatically without being habitual, and automatic processes without a behavioral component are also not considered to reflect habits (e.g., automatic semantic activation). Thus, a crucial feature that characterizes habits on top of their reflecting features of automaticity is that habits refer to operant behaviors that result from some kind of learning or experience.

Importantly, this definition describes what a habit is, but it does not imply specific assumptions regarding its explanation. That is, a habit can be observed regardless of whether the behavior was reinforced in a certain situation or whether it was just executed (repeatedly or just once) in this situation (without necessarily having been reinforced). Relatedly, the definition of habitual behavior is mute with regard to its underlying causes. Habits might reflect associations between situational cues and responses that will emerge gradually as a consequence of repeated and/or rewarded pairings, as early learning theories have assumed. Again, however, alternative conceptions are possible that explain habitual behavior by automatic memory processes, without necessarily drawing on the concept of associations. Whatever the correct theoretical explanation is, characterizing a behavior as habitual implies that it is assumed to share some features of automaticity (e.g., goal-independence, efficiency, speed, unawareness; Bargh, 1994; Moors and De Houwer, 2006), that it is categorized as operant behavior, and that it is somehow related to learning/experience.^1^

The present study proposes an alternative view according to which habit acquisition can be explained by recent cognitive accounts of automatic action regulation that draw on episodic memory models (indeed, this view is also suggested by Wood and Rünger, 2016). In line with such a perspective, we propose the “law of recency” as another route to habitual behavior. According to this instance-based account of habit acquisition, having executed a behavior in a specific situation increases the likelihood of executing the same behavior in the same situation again when it is encountered the next time, even in the absence of reward and although the behavior was executed only once (i.e., in the absence of multiple repetitions). The core focus of our study is to provide a test of the law of recency, and to dissociate influences of an instance-based retrieval of the behavior that was executed during the last encounter with the current situation from alternative explanations in terms of multiple repetitions (global contingencies) and reward. Specifically, we investigate whether habitual behavior resulting from pairings between a stimulus and a response can be explained in terms of such an episodic retrieval of responses. To provide a pure test of habitual behavior resulting from previous pairings, we used a paradigm that does not contain any kind of rewards, thus effectively ruling out any influence of reinforcement on the emergence of habits in our study.

It is important to note that our study does not claim to show that reinforcement is irrelevant for the emergence of habits. We just want to limit our study to the investigation of mere repetition effects, without making any claims regarding the validity of the “law of effect” or its underlying causes. Even if we fully succeeded in explaining effects of practice on the basis of episodic response retrieval, this would still leave room for the possibility of reinforcement having an independent, additional effect on habit acquisition, which may or may not be mediated by episodic retrieval.

### Episodic Memory Models of Automatic, Stimulus-Based Action Regulation

The idea of stimulus-response bindings (“event files,” Hommel, 1998) is a central characteristic for stimulus-based action regulation accounts (Logan, 1988; Hommel et al., 2001; Rothermund et al., 2005). Accordingly, whenever a response is executed to a stimulus, their mental codes become integrated, resulting in episodic stimulus-response bindings that are stored in memory. Stimulus repetition on a later occasion triggers retrieval of the response that was bound to the stimulus. This will facilitate or impede performance, depending on whether the retrieved response is appropriate or not on the current trial. To date, a burgeoning amount of findings attests that storage and retrieval of these episodic stimulus-response bindings are pervasive principles of action regulation and apply to a broad scope of stimuli and responses (for an overview, see Henson et al., 2014).

A crucial difference between stimulus-response bindings and stimulus-response associations in standard learning paradigms is that stimuli and responses are typically not correlated in designs which are used to investigate stimulus-response binding and retrieval (SRBR) effects. Specifically, SRBR effects are assessed in a sequential trial design, in which the factors Stimulus Relation (i.e., does the stimulus repeat or change from trial n-1 to trial n) and Response Relation (i.e., does the response repeat or change from trial n-1 to trial n) are orthogonally manipulated. In other words, there simply is *nothing to learn* over the course of the experiment in these tasks, since each word is presented equally often with each response. Yet, it is an unresolved issue how SRBR effects relate to learning effects. Although this is a much debated and discussed topic, empirical findings so far are scarce and unsystematic (Colzato et al., 2006; Herwig and Waszak, 2012; Moeller and Frings, 2014, 2017; Schmidt et al., 2016, 2019). Some of these studies suggest that SRBR effects are only a transient “by-product” of distributed processing and intentional action planning but are unrelated to persistent learning effects (Colzato et al., 2006; Herwig and Waszak, 2012; Moeller and Frings, 2014, 2017). In turn, other studies favor the view that short-term binding effects and more persistent learning effects are essentially the same thing, only studied at different time scales (Schmidt et al., 2019). Hence, one could conceive of SRBR effects as “one trial learning” that serves as a founding stone for contingent associations which are stored in memory on a long-term basis. This reasoning is further supported by recent computational modeling simulations (Schmidt et al., 2016) which indicate that both types of effects might result from the same underlying learning mechanism.

### An Episodic Account of Habit Acquisition

According to the present account, habitual responding results from (a) storing stimulus-response bindings in memory and (b) retrieving the most recent of these bindings when the stimulus is re-encountered on a later occasion. This leads to a reactivation of the response that was bound to the stimulus during the last occurrence of the stimulus. In other words, habitual responding can be understood as a result of previous stimulus-response bindings that emerged over the course of the experiment. First and foremost, we propose this account – the “law of recency” – as an explanation for habits that are based on repetition. According to this account, it is always the most recent instance of the current stimulus situation that is retrieved on the next occasion, and that influences responding in the current situation via a retrieval of the response that was shown during the previous instance. Our account provides an alternative explanation of repetition effects that competes with association- or frequency-based accounts of repetition-based habits that were proposed in the tradition of the law of exercise (e.g., Miller et al., 2019). The crucial difference between the two accounts is that according to the law of recency, it is the most recent episode that drives responding, whereas according to the law of exercise, the global frequency or contingency of responding to all previous occurrences of this situation is the decisive factor. To distinguish between these accounts, the behavior that was shown during the last occurrence has to be manipulated independently of the global context in which this behavior has been shown.^2^ In the current study, we will manipulate these two factors independently.

Importantly, and in contrast to existing accounts on habit formation, stimulus-response bindings can emerge even in the absence of past reinforcement and hence do not rely on any behavior-reward correlation. Hence, our account predicts that habit formation should be possible even though responses are never reinforced. Importantly, our study is not meant to rule out any effects of reinforcement on habit acquisition (“law of effect”), nor do we test whether any such effect is due to episodic retrieval processes. We just wanted to make sure that the habitual behavior we studied reflects pure repetition effects, which is why we studied behavior in the absence of any tangible rewards.

To test the underlying causes of habit formation in the absence of reinforcement, we used a modified color-word contingency learning (CL) paradigm (e.g., Schmidt et al., 2007; for a review, see MacLeod, 2019). In our task, participants classify the color of printed words (neutral adjectives) on each trial. However, each word is presented most often in two of four colors (high contingency combinations) and less often in the remaining two colors (low contingency combinations, see Table 1). Although the word meaning is irrelevant for the color categorization task, participants learn the contingencies between word stimuli and color responses. Learning of contingencies served as an index of habit formation and is reflected in faster and more accurate performance on high compared with low contingency combinations (Schmidt et al., 2007; for related work, see Miller, 1987; Carlson and Flowers, 1996).

**TABLE 1:** Example for word-color contingency manipulation in Experiments 1 and 2.

	**Color responses**	**Word stimuli**			
		**‘warm’**	**‘klein’**	**‘ganz’**	**‘fast’**
Exp 1	Red	2(*hc*)	2(*hc*)	1(*lc*)	1(*lc*)
	Green	2(*hc*)	1(*lc*)	2(*hc*)	1(*lc*)
	Blue	1(*lc*)	2(*hc*)	1(*lc*)	2(*hc*)
	Yellow	1(*lc*)	1(*lc*)	2(*hc*)	2(*hc*)
Exp 2	Red	4(*hc*)	4(*hc*)	1(*lc*)	1(*lc*)
	Green	4(*hc*)	1(*lc*)	4(*hc*)	1(*lc*)
	Blue	1(*lc*)	4(*hc*)	1(*lc*)	4(*hc*)
	yellow	1(*lc*)	1(*lc*)	4(*hc*)	4(*hc*)

*Exp, Experiment. Hc, high contingency word-color response combinations; lc, low contingency word-color response combinations.*

Deviating from previous research on CL, we chose to study the effects of comparatively weak and complex contingencies on behavior. Previous research already showed that participants produce contingency effects even when unaware of the contingencies, thus establishing the automatic (i.e., habitual) nature of behavior that is driven by the CL (Schmidt et al., 2007). Furthermore, learning in this paradigm is incidental, as participants are not informed in advance of contingencies and the words are irrelevant to the main task of color identification. In our study, we used much weaker contingencies than in the original paradigm, and we employed more complex rules in which one stimulus was systematically paired with two instead of just one response. Through these measures, the contingencies in our study were more subtle and much harder to detect, and they could not be translated into simple S→R rules (due to the dual response pairings), making it even less likely that our participants would be able to use the contingencies strategically. By implication, any effect of CL in our study can be taken as evidence for automatic behavior regulation, thus representing an index of habitual responding.

The core idea of our study is that habit acquisition that is based on CL can be explained in terms of an episodic retrieval of previous stimulus-response episodes (cf. Schmidt et al., submitted). For high contingency trials, probabilities are above chance (which is *p* = 0.25 in a four color choice task) that the word of the current trial was presented in the same color also during its last occurrence (in our study, this probability is *p* = 0.33 and *p* = 0.40 for Experiments 1 and 2, respectively), whereas for low contingency trials, probabilities of word-color repetitions are lower than chance (*p* = 0.17 and *p* = 0.10 for Experiments 1 and 2, respectively). By implication, retrieving the response that was stored together with the word during its last occurrence will facilitate responding for 33% (Experiment 1) or 40% (Experiment 2) of the high contingency trials, but for only 17% (Experiment 1) or 10% (Experiment 2) of the low contingency trials. Likewise, response retrieval of the last episode in which the word was presented will activate a different response and will delay responding for 67% (Experiment 1) or 60% (Experiment 2) of the high contingency trials but for 83% (Experiment 1) or 90% (Experiment 2) of the low contingency trials. Our study aims to test the hypothesis that retrieving the response from the last occurrence of the word stimulus drives the CL effect, and is the underlying mechanism of habit formation. We predicted that controlling for these differences in retrieving either the same or a different response should eliminate the global CL effect (cf. Schmidt et al., 2019).

As a crucial design feature of our study, we aimed to assess episodic response retrieval effects and CL effects *simultaneously*, that is, in the very same experiment. Our study had the following expectations: First, we predicted to find robust CL effects. Second, we predicted to find response retrieval effects (reflected in an effect of response relation regarding the current and previous occurrence of the word). Third, and most central to our research aims, we tested whether response retrieval effects can explain habit formation (i.e., the CL effect). We expected that CL will be substantially reduced (or even eliminated) as soon as we control for differences in response retrieval effects. Such a pattern of results would support the law of recency as an explanation of habitual behavior, while at same time controlling for (and ruling out) an alternative explanation in terms of the law of exercise (i.e., a global, frequency based account of repetition effects).

## Experiment 1

### Method

#### Participants

Thirty native German-speaking FSU Jena students (18 female; *M*_age_ = 23.03 years; range: 18–30 years) took part in the experiment. *A priori* power calculations (G^∗^Power 3; Faul et al., 2007) showed that we need at least 27 participants to detect a medium sized effects (*d* = 0.5) with sufficient power (1-β ≥ 0.8). Up to six participants were tested in parallel. Each participant was seated individually in a small cubicle. Sessions lasted 25 min. Participants received €2.50 for their participation plus a chocolate bar or ice cream voucher if they fulfilled criteria for speed (more than 80% of all reaction times [RT] faster than 1000 ms) and accuracy (less than 15% errors) in the experimental trials. In accordance with guidelines of the American Psychological Association, prior to the study, all participants gave their explicit consent to take part via pressing the “j” key of the keyboard (responses to the informed consent were saved for each participant). The study was canceled before any data collection started for participants who did not give their consent. An ethics approval was not required as per applicable institutional and national guidelines and regulations because no cover-story or otherwise misleading or suggestive information was conveyed to participants (this procedure is in accordance with the ethical standards at the Institute of Psychology of the FSU Jena).

#### Apparatus and Stimuli

The experiment was programed with E-Prime 3.0. Stimuli were the four neutral monosyllabic German adjectives “warm” (“warm”), “klein” (“small”), “ganz” (“whole”) and “fast” (“almost”). Stimuli were presented in Times New Roman font (16 pts.) on a black background on a 17′′ inch CRT screen. A response pad, attached to the computer via the parallel port, served to collect responses. Participants responded by pressing four colored keys on the response pad with their middle and index fingers of the left and right hand (key order from left middle to right middle finger: red, green, blue, yellow). A fifth key, operated via (left or right) thumb press, was labeled with “Los” (“go”) and served to start the experiment.

#### Design

Central to our study, we manipulated the contingency between word stimuli and color responses: Each of the four word stimuli appeared in each of the four colors; however, combinations differed in their frequencies. Specifically, each word appeared twice as often in two colors (high contingency combinations) than in the two remaining colors (low contingency combinations), yielding a contingency ratio of 2:1. Thus, each word was predictive of two colors/responses (high contingency combinations) and non-predictive of the other two colors/responses (low contingency combinations).^3^

The contingency manipulation resulted in 16 different word-color combinations. Given that high contingency combinations were shown twice as often as low contingency combinations, this amounted to a total of 24 word-color combinations (i.e., 16 word-color combinations plus 8 “duplicates” resulting from the 2:1 contingency manipulation, see Table 1). Each word-color combination was presented as stimulus in trial n-1 and as stimulus in trial n, resulting in a total of 24^∗^24 = 576 experimental trials.

As another advantage, the chosen design allowed us to analyze immediate trial sequences to assess SRBR effects in a systematic and fully controlled manner. For immediate trial sequences within each experimental list, we realized a maximally balanced 2 (contingency of present trial n: high vs. low) × 2 (contingency of preceding trial n-1: high vs. low) × Stimulus Relation between trial n and trial n-1 (stimulus repetition [SR; 25%] vs. stimulus change [SC; 75%]) × Response Relation between trial n and trial n-1 (response repetition [RR; 25%] vs. response change [RC; 75%]) design. Note that trial sequences for the SR-RR cell are only possible when trial n-1 and trial n both represent high contingency trials or when both represent low contingency trials (i.e., when the contingency *matches* between a trial sequence). Put differently, if both the stimulus and the response repeat in a given trial n from the previous trial n-1, then the contingency from the trial n-1 has to repeat as well. In turn, contingency *mismatches* (e.g., high contingency on trial n-1, but low contingency on trial n, or vice versa) are impossible to create within the SR-RR cell. Thus, to analyze SRBR effects, only trial sequences with matching contingencies were regarded.

#### Procedure

Instructions were given on screen. Participants were informed that on every trial, a word stimulus would first appear in white font and then change its color to red, green, blue, or yellow. Their task was to categorize the color of each word stimulus by pressing the corresponding key on the response pad. After reading the instructions, participants worked through 24 practice trials that were identical to trials in the experimental blocks. The practice block was repeated if more than 20% errors were committed. If error rates still exceeded 20% after the third run of the practice block, the experiment was terminated (however, this never occurred during data collection). Upon successful completion of the practice block, the main experiment started, consisting of 576 experimental plus 1 filler trial (i.e., trial 1, which had no preceding trial). After 288 trials were completed, participants were given a small, self-paced break. The first trial after the break was identical to the last trial before the break and served as filler. Filler trials were not analyzed. Experimental trials were presented in a continuous fashion. At the end of the experiment, participants were rewarded accordingly.

Each trial started with a fixation cross (500 ms), followed by a white word for a variable duration by randomly selecting one out of five possible durations (150, 200, 250, 300, or 350 ms) after which the word changed its color until key press. Erroneous responses elicited the feedback message “Fehler – reagiere sorgfältiger! Weiter mit ‘Los’ Taste” (“Error – be more accurate! Continue with ‘go’ key…”). Responses slower than 1000 ms elicited the feedback message “Zu langsam – reagiere schneller! Weiter mit ‘Los’ Taste” (Too slow – respond faster! Continue with ‘go’ key…”). Feedback was displayed in white font on red background until key press. Then, the next trial started.

### Results

Trials with erroneous responses (6.8%) and RT outliers^4^ (2.6%) were excluded from all analyses.

#### Contingency Learning Effects

We compared performance in low contingency (*M*_RT_ = 534ms; *M*_err_ = 6.7%) with high contingency trials (*M*_RT_ = 528ms; *M*_err_ = 6.9%). For RTs, this comparison yielded a significant CL effect of Δ_low__–__high_ = 6 ms, *t*(29) = 3.13, *p* = 0.004, *d*_z_ = 0.57, BF_10_ = 9.08. For error rates, the effect was not significant (Δ_low__–__high_ = −0.2%, |*t*| < 1).

#### Explaining Contingency Learning Effects by Response Retrieval Effects

**FIGURE 1:**
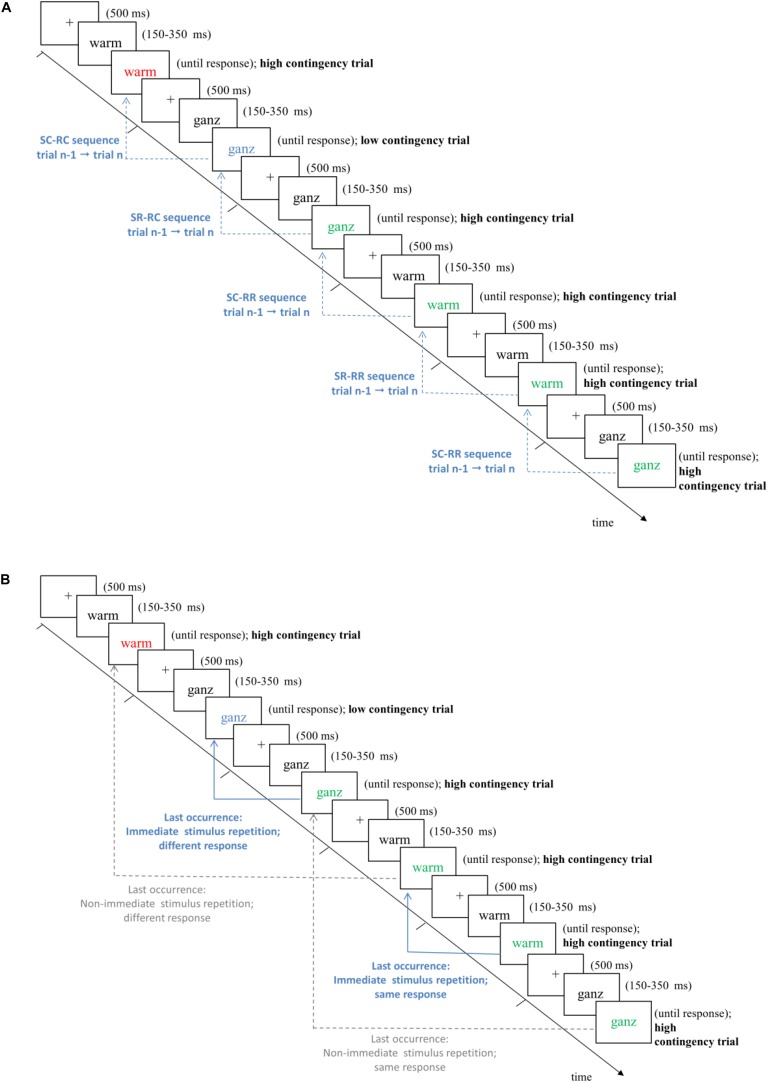
Schematic trial procedure in Experiments 1 and 2. Note that in the experiments, all stimuli were presented on black background in white font or in the respective colors (see Table 1). For both figures, we inverted the coloring scheme only for illustrative purposes. Stimuli are not drawn to scale. Trials are classified as high vs. low contingency trials (for details, see Table 1). Arrows in **(A)** illustrate different trial types for immediate sequence effects from trial n-1 to trial n to test for immediate SRBR effects (SR, stimulus repetition; SC, stimulus change; RR, response repetition; RC, response change). Arrows in **(B)** illustrate trial classification for the central analyses of interest to explain contingency learning effects by response retrieval effects, i.e., whether a given trial reflected an immediate (solid/blue lines) vs. non-immediate (dotted/gray lines) stimulus repetition trial (factor Distance) with same or different response (factor Previous Response) compared to the last occurrence of the stimulus word. See main text for details.

We investigated whether response retrieval effects influenced responding, and whether they can explain CL effects. To this end, every trial was referenced back to the *last prior occurrence* of the current stimulus – effectively, this implies that this analysis is based on stimulus repetitions (see Figure 1B). Furthermore, stimulus repetition trials were coded with regard to two additional factors: First, we coded the relation between the responses to the word in the current trial as well as during its last occurrence, which could be the same or different (factor Previous Response). Second, we coded how distant the last occurrence was from the present stimulus repetition trial (factor Distance: immediate vs. non-immediate stimulus repetition). Distance was coded as a binary factor with “immediate stimulus repetition” indicating that the present stimulus was repeated from the immediately preceding trial n-1. In turn, trials in which the last occurrence of the current word stimulus were further away (i.e., trials n-2 to n-30) were coded as “non-immediate stimulus repetition” (see Figure 1B for illustrations). Only last occurrences in which a correct response was committed were included. Thus, data were analyzed in a 2 (contingency: high vs. low) × 2 (previous response: same vs. different) × 2 (distance: immediate vs. non-immediate stimulus repetition) ANOVA on mean RTs (the pattern of means is shown in Table 3).

Although we obtained a significant CL effect in our first analysis (without controlling for SRBR effects, see above), the main effect of contingency was no longer significant in the final analysis, *F* < 1, BF_01_ = 6.79. Instead, the ANOVA yielded a main effect of previous response, *F*(1,29) = 179.96, *p* < 0.001, η_*p*_^2^ = 0.86, BF_10_ = 3.817e + 21, indicating that performance was faster if the current stimulus repetition required the same previous response (*M* = 480 ms) compared with a different previous response (*M* = 548 ms). This pattern of findings confirms our hypothesis that controlling for episodic SRBR effects effectively eliminated the CL effect in Experiment 1. The main effect of the distance factor was also significant, *F*(1,29) = 141.22, *p* < 0.001, η_*p*_^2^ = 0.83, indicating that performance was generally faster for immediate stimulus repetitions (*M* = 497 ms) compared to trials in which the last occurrence of the same word stimulus was more distant (*M* = 531 ms). Main effects were qualified by a Distance × Previous Response interaction, *F*(1,29) = 322.52, *p* < 0.001, η_*p*_^2^ = 0.92. Follow-up tests showed that response retrieval effects were significantly stronger for immediate stimulus repetitions (*M*_sameresponse_ = 432 ms; *M*_differentresponse_ = 562 ms; *t*[29] = 16.53, *p* < 0.001, *d*_z_ = 2.78), but were also significant for stimulus repetitions of more distant trials (*M*_sameresponse_ = 528 ms; *M*_differentresponse_ = 534 ms; *t*[29] = 1.76, *p* = 0.045, one-tailed, *d*_z_ = 0.32). No other effect was significant (all *F*s < 2.9, all *p*s ≥ 0.10).

#### Multi-Level Analyses

We also conducted multi-level analyses on the basis of individual trials, treating trials as nested within subjects. In these analyses, CL and response retrieval reflect between factors (on the level of trials), which allows us to simulate a stepwise regression approach to test whether entering response retrieval as an additional predictor in a second step eliminates effects of CL that had been significant when entered as a single predictor into the regression equation in step 1. The multi-level analyses also allow us to treat distance of the last occurrence as a continuous predictor, so we can calculate at which distance the effect of response retrieval effectively becomes zero.

A multilevel analysis with contingency (high frequency = 1 vs. low frequency = 2) as the only level 1 predictor, allowing for random intercepts and slopes, yields a significant CL effect, β = 6.19, *t* = 3.15, *p* = 0.004, replicating the effect of the previous analysis. Adding Previous Response (same = 1 vs. different = 2), as an additional level 1 predictor in a second step produced a highly significant effect for this variable, β = 34.21, *t* = 9.30, *p* < 0.001, and it rendered the effect for the CL variable non-significant, β = 0.59, *t* = 0.28, *p* = 0.78. Effectively, then, although CL predicts RT when considered in isolation, this effect is fully explained by response retrieval.

Although we were primarily interested in the main effects of CL and response retrieval, the multinomial model also allows us to introduce an interaction term for the two variables (CL × previous response). Adding the product term in a third step yields a beta that is positive and significant (*t* = 2.19, *p* = 0.029). This interaction indicates that effects of response retrieval were slightly stronger for low contingency trials, that is, responses were slowest for low contingency trials in the “different response” condition. A plausible explanation for this asymmetry is that response retrieval may not only be influenced by the last occurrence of the stimulus but may probably also sometimes retrieve an earlier episode in which the stimulus was presented. For low contingency trials in the “different response” condition, such a retrieval of an earlier episode will retrieve a different response in 83% of these trials. For high contingency trials in the different response condition, only 67% of the previous occurrences of the word contained a different response, 33% of the trials contained an identical response. It is thus possible that in some high contingency trials in the “different response” condition, the correct response was retrieved from an earlier episode (leading to a facilitative effect that counteracted the delay effect in the “different response” condition), even though the last occurrence of the word was paired with a different response.

Another multi-level analysis was used to evaluate the moderating effect of distance on effects of response retrieval. For this purpose, we predicted RT with the previous response factor (pr), distance (d), and their interaction (pr × d). We also added a squared term for distance (*d*^2^) and the interaction of this term with previous response (pr × d^2^) to allow for a non-linear decline of the influence of response retrieval with increasing distance. The full model yielded significant effects for all predictors (all *p* < 0.001). The regression equation is given by the following set of parameter values: RT = 341 + 105.31pr + 46.72d–2.11d^2^–25.43pr × d + 1.15pr × d^2^. Transforming this equation into a form that represents the slope of pr as function of *d* and *d*^2^ gives: RT = 341 + (105.31–25.43d + 1.15d^2^)^∗^pr + 46.72d–2.11d^2^. Setting the quadratic formula in brackets that represents the slope for pr to zero and solving for *d* yields *d* = 5.52, that is, the slope for response retrieval becomes zero at a distance between 5 and 6 trials.

#### Stimulus-Response Binding and Retrieval Effects

To test for SRBR effects, we analyzed immediate sequence effects from trial n-1 to trial n (cf. Figure 1A). In these analyses, only sequences with matching contingencies were regarded (see Method section for details). We performed two separate 2 × 2 × 2 repeated measurement analyses of variance (ANOVA) with the factors stimulus relation (stimulus repetition vs. stimulus change from trial n-1 to trial n), response relation (response repetition vs. response change from trial n-1 to trial n), and type of prime-probe contingency match (both trial n-1 and trial n high contingency vs. both low contingency) on trial n performance (i.e., RTs and error rates; see Table 2 for means).

**TABLE 2:** Results for SRBR effects (probe RT and error rates) in Experiments 1 and 2.

				**Probe RT (ms)**		**Probe errors (%)**	
	**Type of contingency match**	**Response relation**	**Stimulus relation**	***M***	***SD***	***M***	***SD***
	**trial n-1 → trial n**	**trial n-1 → trial n**	**trial n-1 → trial n**				
Exp 1	High–high	RR	SR	432	40	1.8	3.3
			SC	456	41	3.9	4.7
		RC	SR	559	58	7.5	4.1
			SC	548	50	8.1	4.8
	Low–low	RR	SR	426	45	1.4	4.2
			SC	460	38	2.6	5.3
		RC	SR	562	60	6.5	9.5
			SC	554	49	7.6	4.7
Exp 2	High–high	RR	SR	409	33	4.2	4.7
			SC	452	38	5.5	4.4
		RC	SR	537	53	9.3	6.0
			SC	529	52	8.5	5.6
	Low–low	RR	SR	415	50	3.5	7.0
			SC	451	61	7.0	12.6
		RC	SR	553	77	13.3	19.2
			SC	551	54	11.1	11.0

*Exp, Experiment. RR, response repetition. RC, response change. SR, stimulus repetition. SC, stimulus change.*

For RTs, the ANOVA yielded significant main effects of stimulus relation, *F*(1,29) = 7.74, *p* = 0.009, η_*p*_^2^ = 0.21, and response relation, *F*(1,29) = 174.16, *p* < 0.001, η_*p*_^2^ = 0.86, indicating that RTs were faster for stimulus repetition (*M* = 495 ms) compared with stimulus change trials (*M* = 505 ms) and that probe RTs were faster for response repetitions (*M* = 444 ms) than for response changes (*M* = 556 ms). Most importantly, both effects were qualified by a significant Stimulus Relation × Response Relation interaction, *F*(1,29) = 39.62, *p* < 0.001, η_*p*_^2^ = 0.58, that reflected the typical pattern of SRBR effects. Follow-up tests showed that compared to stimulus change from trial n-1 to trial n, stimulus repetition significantly sped up performance by Δ_SCRR–SRRR_ = 29ms, *t*(29) = 5.48, *p* < 0.001, *d*_z_ = 1.00, for response repetition. In turn, stimulus repetition (compared with stimulus change from trial n-1 to n) significantly slowed down performance by Δ_SCRC–SRRC_ = −10 ms, *t*(29) = 2.42, *p* = 0.022, *d*_z_ = 0.44, for response changes. No other effect was significant (all *F*s < 1.06, all *p*s > 0.30).

For error rates, the same ANOVA yielded only a main effect of response relation, *F*(1,29) = 65.81, *p* < 0.001, η_*p*_^2^ = 0.69, indicating that participants made fewer errors on response repetition (*M* = 2.4%) than on response change sequences (*M* = 7.4%). No other effect was significant (all *F*s < 3.2, all *p*s > 0.08).

### Discussion

The results of Experiment 1 are clear-cut: First, we obtained a CL effect, indicating that participants incidentally learned the word-color response associations over the course of the experiment. Second, we obtained robust response retrieval effects, reflecting faster RTs in the current trial when the same response had been given during the last occurrence of the word stimulus that was also presented in the current trial, compared to trials when a different response had been executed during the last occurrence. Third and most central to our research aims, the CL effect was effectively eliminated after controlling for effects of response retrieval. This pattern of findings emerged both for ANOVA analyses with aggregated data and also in multilevel analyses in which CL and response retrieval were coded on a trial level. Importantly, effects of response retrieval were not limited to the immediately preceding trial, but were found for distances up to 5–6 trials, ruling out alternative explanations of the effect in terms of mere response repetition. For immediate stimulus repetition sequences (distance = 1), effects of response retrieval are identical to effects of response repetition, until sequences in which the stimulus changes are used as a baseline. These analyses replicated the standard pattern of SRBR effects that obtained in many previous studies (Rothermund et al., 2005; see also Frings et al., 2007; Giesen and Rothermund, 2014), rendering explanations of response retrieval effects in terms of mere response repetition unlikely. Together, findings from Experiment 1 support predictions derived from the law of recency that episodic retrieval of responses from the most recent occurrence of the stimulus represents a central process underlying habit formation (i.e., learning of word-response contingencies). Effects of global SR contingencies were completely eliminated after controlling for an influence of the most recent last episode, which rules out frequency-based explanations (law of exercise) of habitual responding in the current study.

The CL effect observed in Experiment 1 was smaller than in previous studies [*d*_z_ = 0.57, reflecting a medium-sized effect according to Cohen (1969) compared with effect sizes between *d*_z_ = 0.62 up to *d*_z_ = 1.24, reflecting medium-to-large- to very-large-sized effects in Schmidt et al., 2007]. In our view, this is probably due to the fact that Experiment 1 had a contingency ratio of only 2:1, which is a rather weak contingency manipulation in and of itself and it is known that the magnitude of contingency effects is proportional to the contingency (Forrin and MacLeod, 2018; see also, Schmidt and De Houwer, 2016). The low contingency was chosen on purpose, since we wanted to make sure that contingencies went undetected, and thus could not be applied in a strategic fashion. However, being aware of the fact that single studies pose the risk of being unreliable (Cesario, 2014; see also Tversky and Kahneman, 1971) and that replication is an increasingly important research value (Nosek et al., 2012), we ran a second experiment with the aim to replicate our initial findings from Experiment 1, but with a stronger contingency manipulation (ratio of 4:1) to boost CL effects. By increasing the contingency we wanted to establish that the contingency effect itself is strong beyond any reasonable doubt, so that eliminating the effect by controlling for effects of response retrieval cannot be attributed to the contingency effect being unreliable in the first place. Although the contingency that was chosen in Experiment 2 is stronger than in Experiment 1, we want to emphasize that it is still much weaker than in previous studies that already demonstrated contingency effects in the absence of awareness (Schmidt et al., 2007). Furthermore, Experiment 2 again used contingencies in which one stimulus was predictive of two different responses, preventing a simple strategic use of the contingencies for response preparation. Furthermore, Experiment 2 was preregistered online before any data collection started (see details below).

## Experiment 2

### Method

#### Participants

Forty native German-speaking FSU Jena students (27 female; *M*_age_ = 23.3 years, range: 18–32 years) took part in the experiment. We decided to recruit a somewhat larger number of participants compared to Experiment 1 in order to be able to detect effects of CL that are even smaller than medium in size (*d* = 0.4) with sufficient power (1-β ≥ 0.8). Power calculations were conducted with G^∗^Power 3 (Faul et al., 2007). All participants gave their explicit verbal consent to take part prior to the study. Session duration and payment of participants were similar to Experiment 1.

#### Apparatus, Stimulus, Design, and Procedure

Apparatus, stimuli, design, and procedure were similar to Experiment 1 except for the following changes. In Experiment 2, we used a stronger contingency manipulation: Each word appeared four times more often in two colors (high contingency combinations) than in the two remaining colors (low contingency combinations, see Table 1), resulting in a contingency ratio of 4:1. As in Experiment 1, each word was predictive of two colors/responses (high contingency combinations), only more strongly in the present experiment, and non-predictive of the other two colors/responses (low contingency combinations).

The contingency manipulation resulted in 16 different word-color combinations plus 24 “duplicates” resulting from the 4:1 contingency manipulation, thus amounting to a total of 40 word-color combinations. To control of immediate sequences, each word-color combination was then presented as stimulus in trial n-1 and as stimulus in trial n, yielding a total of 40^∗^40 = 1600 experimental trials. Since this number of experimental trials would have resulted in an experiment of unreasonable length, the total list was always split among a group of three participants, taking care that the orthogonal variation of stimulus relation and response relation was maintained for each participant. This resulted in 535 experimental trials + 1 filler trial per participant. Procedural details were again similar to Experiment 1, with the only exception that whenever a timing or response error was committed, participants had to press the correct response key (instead of the “go” key) to continue the experiment.

#### Preregistration

Prior to data collection, we preregistered the exact method, design, hypotheses, data preparation, and planned data analyses online at www.aspredicted.org.^5^

### Results

According to the same criteria as in Experiment 1, trials with erroneous responses (8.1%) and RT outliers (3.0%) were excluded from all analyses.

#### Contingency Learning Effects

We compared performance in low contingency (*M*_RT_ = 517 ms; *M*_err_ = 9.4%) with high contingency trials (*M*_RT_ = 508 ms; *M*_err_ = 7.7%). These comparisons yielded significant CL effects for RTs, Δ_low__–__high_ = 9 ms, *t*(39) = 4.41, *p* < 0.001, *d*_z_ = 0.70, BF_10_ = 242.99, and also for error rates, Δ_low__–__high_ = 1.6%, *t*(39) = 2.83, *p* = 0.007, *d*_z_ = 0.45, BF_10_ = 5.64.

#### Explaining Contingency Learning Effects by SRBR Effects

We investigated retrieval effects and whether the CL effect is reduced or eliminated as soon as we control for these effects, following the same approach as in Experiment 1. Thus, we performed a 2 (probe contingency: high vs. low) × 2 (previous response: same vs. different) × 2 (distance: immediate vs. non-immediate stimulus repetition) ANOVA on mean RTs (the pattern of means for this analysis is shown in Table 3).

**TABLE 3:** Average RTs (and SDs) for the combinations of contingency (high vs. low), previous response (same vs. different), and distance (immediate vs. non immediate stimulus repetition) in Experiments 1 and 2.

				**RT (ms)**	
	**Contingency**	**Distance**	**Previous**	***M***	***SD***
			**response**		
Exp 1	High	Immediate stimulus	Same	432	40
		repetition	Different	560	52
		Non-immediate	Same	533	46
		stimulus repetition	Different	533	45
	Low	Immediate stimulus	Same	432	49
		repetition	Different	564	47
		Non-immediate	Same	522	39
		stimulus repetition	Different	536	46
Exp 2	High	Immediate stimulus	Same	409	33
		repetition	Different	537	51
		Non-immediate	Same	500	44
		stimulus repetition	Different	516	44
	Low	Immediate stimulus	Same	415	50
		repetition	Different	559	60
		Non-immediate	Same	482	74
		stimulus repetition	Different	520	44

*Exp, Experiment.*

Importantly, the main effect of contingency was no longer significant in this analysis, *F*(1,39) = 1.34, *p* = 0.254, η_*p*_^2^ = 0.03, BF_01_ = 7.26. Furthermore, the ANOVA yielded additional main effects of previous response, *F*(1,39) = 276.64, *p* < 0.001, η_*p*_^2^ = 0.88, BF_10_ = 1.078e + 38, indicating that performance was faster if the current stimulus repetition trial required the same previous response (*M* = 452 ms) compared with a different previous response (*M* = 533 ms). These findings replicate Experiment 1 and show that controlling for response retrieval effects effectively eliminated the CL effect also in Experiment 2. There was also a main effect of distance, *F*(1,39) = 64.04, *p* < 0.001, η_*p*_^2^ = 0.62, meaning that performance was faster if the stimulus was repeated from the immediately preceding trial n-1 (*M* = 480 ms) compared with non-immediate stimulus repetitions from more distant trials (*M* = 504 ms).^6^ Both main effects were again qualified by a significant Distance × Previous Response interaction, *F*(1,39) = 198.75, *p* < 0.001, η_*p*_^2^ = 0.84. Follow-up tests showed that response retrieval effects were stronger for immediate stimulus repetitions (*M*_sameresponse_ = 412 ms; *M*_differentresponse_ = 548 ms; *t*[39] = 19.07, *p* < 0.001, *d*_z_ = 3.01), but were still significant for stimulus repetitions of more distant trials (*M*_sameresponse_ = 491 ms; *M*_differentresponse_ = 518 ms; *t*[39] = 5.16, *p* < 0.001, *d*_z_ = 0.82). Two additional interactions were significant: First, the Distance × Contingency interaction, *F*(1,39) = 11.95, *p* = 0.001, η_*p*_^2^ = 0.24. Follow-up tests revealed that distance had a stronger facilitating effect for the high contingency trials (*M*_immediate_ = 473 ms, *M*_non–immediate_ = 508 ms, *t*[39] = 12.81, *p* < 0.001, *d*_z_ = 2.03) than for the low contingency trials (*M*_immediate_ = 487 ms, *M*_non–immediate_ = 501 ms, *t*[39] = 2.55, *p* = 0.015, *d*_z_ = 0.40). Second, the interaction between Previous Response × Contingency was significant as well, *F*(1,39) = 7.79, *p* = 0.008, η_*p*_^2^ = 0.17. Follow-up tests showed that response retrieval effects were stronger for high contingency trials (*M*_sameresponse_ = 454 ms; *M*_differentresponse_ = 526 ms; *t*[39] = 17.76, *p* < 0.001, *d*_z_ = 2.81) than for low contingency trials (*M*_sameresponse_ = 449 ms; *M*_differentresponse_ = 539 ms; *t*[39] = 12.45, *p* < 0.001, *d*_z_ = 1.97).^7^ No other effect was significant (*F* < 1, *p* > 0.60).

#### Multi-Level Analyses

Like in the previous experiment, we also conducted multi-level analyses on the basis of individual trials, treating trials as nested within subjects. A multilevel analysis with contingency (high frequency = 1 vs. low frequency = 2) as the only level 1 predictor, allowing for random intercepts and slopes, yields a significant CL effect, β = 8.60, *t* = 4.26, *p* < 0.001, replicating the effect of the previous analysis. Adding Previous Response (same = 1 vs. different = 2), as an additional level 1 predictor in a second step produced a highly significant effect for this variable, β = 44.52, *t* = 15.71, *p* < 0.001, and it rendered the effect for the CL variable non-significant, β = 0.33, *t* = 0.16, *p* = 0.87. Effectively, then, although CL predicts RT when considered in isolation, this effect is fully explained by response retrieval.

As in the previous experiment, the product term CL × previous response was significant again (*t* = 7.00, *p* < 0.001). Again, this interaction indicates that effects of response retrieval were slightly stronger for low contingency trials, that is, responses were slowest for low contingency trials in the “different response” condition. For a possible explanation of this interaction effect, see the corresponding paragraph in the results section of Experiment 1.

Another multi-level analysis was used to evaluate the moderating effect of distance on effects of response retrieval. The full model yielded significant effects for all predictors (all *p* ≤ 0.001). The regression equation is given by the following set of parameter values: RT = 299 + 118.22pr + 47.30d–2.26d^2^–26.08pr × d + 1.24pr × d^2^. Transforming this equation into a form that represents the slope of pr as function of *d* and *d*^2^ gives: RT = 299 + (118.22–26.08d + 1.24d^2^)^∗^pr + 47.30d–2.26d^2^. Setting the quadratic formula in brackets that represents the slope for pr to zero and solving for *d* yields *d* = 6.61, that is, the slope for response retrieval becomes zero at a distance between 6 and 7 trials.

#### Stimulus-Response Binding and Retrieval Effects

Like in Experiment 1, when analyzing SRBR effects, only trial n-1 to trial n sequences with matching contingencies were regarded. We performed two separate 2 × 2 × 2 repeated measurement ANOVA with the factors stimulus relation (stimulus repetition vs. stimulus change from trial n-1 to trial n), response relation (response repetition vs. change from trial n-1 to trial n), and type of sequential contingency match (high-high vs. low-low contingency) on performance in trial n (RTs and error rates; see Table 2 for means).

For RTs, the ANOVA yielded significant main effects of contingency type, *F*(1,39) = 9.76, *p* = 0.003, η_*p*_^2^ = 0.20, stimulus relation, *F*(1,39) = 24.04, *p* < 0.001, η_*p*_^2^ = 0.38, and response relation, *F*(1,39) = 222.39, *p* < 0.001, η_*p*_^2^ = 0.85. Accordingly, RTs were faster for high-high contingency trial sequences (*M* = 481 ms) than for low-low contingency trial sequences (*M* = 493 ms); further, RTs were faster for stimulus repetition (*M* = 479 ms) than for stimulus change sequences (*M* = 496 ms); last, RTs were faster for response repetition (*M* = 432 ms) than for response change sequences (*M* = 542 ms). These main effects were qualified by several interactions: Contingency type significantly interacted with response relation, *F*(1,39) = 5.41, *p* = 0.025, η_*p*_^2^ = 0.12 (however, this interaction is not of theoretical interest and is thus not discussed further). Central to our predictions, the Stimulus Relation × Response Relation interaction was also significant, *F*(1,39) = 38.15, *p* < 0.001, η_*p*_^2^ = 0.49. Follow-up tests showed that compared with stimulus changes from trial n-1 to trial n, stimulus repetition significantly sped up performance by Δ_SCRR–SRRR_ = 39 ms, *t*(39) = 7.02, *p* < 0.001, *d*_z_ = 1.10, for response repetition sequences. In turn, stimulus repetition (compared with stimulus changes from trial n-1 to trial n) descriptively slowed down performance by Δ_SCRC–SRRC_ = −5 ms, *t*(39) = 1.19, *p* = 0.24, *d*_z_ = 0.18, for response change sequences. No other effect was significant (all *F*s < 1, all *p*s > 0.36).

For error rates, the same ANOVA yielded only a main effect of response relation, *F*(1,39) = 23.77, *p* < 0.001, η_*p*_^2^ = 0.38, indicating that participants made fewer errors on response repetition (*M* = 5.0%) than on response change sequences (*M* = 10.5%). No other effect was significant (all *F*s < 3.7, all *p*s > 0.062).

### Discussion

In Experiment 2, we used a stronger contingency manipulation to boost CL effects, which was successful. What is more, findings from Experiment 2 fully replicate the pattern of effects that were obtained in Experiment 1. In detail, we obtained a robust CL effect that was larger (*d*_z_ = 0.70) than in Experiment 1 (*d*_z_ = 0.57). Thus, we can conclude that participants incidentally acquired word-response associations over the course of the experiment. Second, we obtained robust response retrieval effects. Third, the CL effect was again effectively eliminated when we controlled for response retrieval. Data from both experiments thus support the law of recency according to which habit formation is mediated by episodic retrieval processes, which lead to a reactivation of the response that was stored in episodic memory together with the stimulus during the most recent occurrence of the current situation. Fourth, as in the previous experiment, the influence of response retrieval was not limited to the previous trial but was visible for distances up to 6–7 trials. Finally, standard SRBR effects for immediate (n-1- > n) sequences in which stimulus changes are used as a baseline condition were replicated also in this experiment, indicating that response retrieval effects cannot be attributed to mere response repetition.

## General Discussion

The present study provides initial evidence that response retrieval effects may fully explain effects of CL (see also Schmidt et al., 2019) and thus provide a potential explanation for learning processes that eventually lead to habitual behavior. In this respect, our study supports the claim that habit formation can be mediated by episodic response retrieval processes regarding the most recent previous occurrence of the current situation (law of recency). These conclusions are supported by data of two experiments, which yielded robust evidence of the following effects: First, participants of both experiments acquired contingencies between stimulus words and color responses over the course of each experiment, leading to faster and more correct responses in trials with high frequency compared to low frequency combinations of word and color. Importantly, participants were never explicitly informed about any contingency relation between words and responses. However, incidental knowledge about the inbuilt word-response contingency was acquired nonetheless and impacted performance, leading to habitual responding. What is more, we obtained these findings even in the absence of any explicit reinforcement schedule (apart from ordinary feedback regarding errors and slow responses that was given on a negligible number of trials). Second, participants in both experiments also displayed episodic binding and retrieval effects, reflected in performance benefits when the word had been presented in the same color during the current trial and the trial in which the word had been presented during its last occurrence, reflecting a stimulus-based retrieval of the response from the previous trial. Third and most importantly, both effect types were not independent: That is, when we controlled for response retrieval effects in joint analyses, the CL effect was effectively eliminated in both experiments. Together, the present findings support the view that episodic binding effects and persistent forms of learning (e.g., habit acquisition) might result from the same underlying learning mechanism (i.e., episodic binding and retrieval). Our findings support the law of recency that explains habit acquisition as an instance-based process. According to this principle, habitual behavior emerges by retrieving and repeating a behavior that was executed during the last encounter with the current situation.

### Theoretical Implications

The present study exemplifies that habit acquisition that is based on CL can be explained in terms of an episodic retrieval of previous stimulus-response episodes (for further recent evidence, see also Schmidt et al., 2019). In this respect, habitual responding can be understood as resulting from the retrieval of stimulus-response bindings that were stored in memory during the last occurrence of the situation that is now encountered again (law of recency).

#### Behavioral Signatures of the Law of Recency

The law of recency has a characteristic behavioral signature that has been demonstrated in numerous studies (and also in the present study) that revealed effects of SRBR. Basically, the core finding attesting to the law of recency consists in an interaction of stimulus relation and response relation in successive trials of a forced-choice reaction task: Repeating the prime stimulus in the probe leads to facilitation for response repetition sequences, but produces interference for response change sequences (Rothermund et al., 2005; see also Hommel, 1998; Mayr and Buchner, 2006; Frings et al., 2007; Giesen et al., 2012; Giesen and Rothermund, 2014). This pattern can be explained by a retrieval and reactivation of the response information of the prime during the probe. The current study demonstrates that SR binding and retrieval also plays a role in a CL paradigm (Schmidt et al., 2007), and – crucially – that effects of episodic SR binding during the last occurrence are what underlies the CL effect.

The law of recency can be used to generate alternative explanations for a wide range of experimental paradigms that investigated effects of global contexts on behavior (e.g., context effects in interference paradigms, Logan and Zbrodoff, 1979). In many of these paradigms, global effects can be tested against local effects of episodic retrieval in order to see whether the law of recency can account for these effects.

Comparing the current study with a large literature on habit acquisition addressing habit formation mostly in animals reveals a crucial difference: Classical studies typically focus on response frequencies as an outcome variable, whereas our study used performance indices (response speed and accuracy) as dependent variables. Relatedly, our study used a forced-choice color categorization task, whereas standard studies focus on a single qualitative response, the frequency of which is counted (e.g., lever pressing). The paradigm that is chosen to investigate habits may influence the results, so it is perhaps hard to compare findings across these very different experimental approaches. Despite its dissimilarity to the paradigms that were typically employed in habit research in the animal literature, focusing on RTs (instead of response frequencies) may offer some advantages for the study of habitual behavior in humans. It is no coincidence that implicit measures aiming at assessing, for instance, implicit prejudice or stereotypes typically rely on RT measures (e.g., Wittenbrink and Schwarz, 2007; Gawronski and Payne, 2010; Klauer et al., 2012). The reason is that the speed of responding is much less controllable than the execution of a specific response, and thus provides a “window to the mind” and into automatic influences of human behavior (see Wentura and Rothermund, 2007).

#### Mathematical Modeling Approaches to the Law of Recency

Processes of an instance-based retrieval of previous stimulus-response episodes have been modeled mathematically within the Parallel Episodic Processing model (PEP; Schmidt et al., 2016). The model is specialized to simulate RT and error data in speeded response time tasks and has been shown to be a powerful tool that successfully simulates and explains experimental findings across a wide variety of experimental paradigms with just one mathematical architecture. For details regarding the modeling approach, we refer the reader to the original articles in which the PEP is presented (e.g., Schmidt et al., 2016). Although the PEP has been developed to account for RT data, its basic rationale might also be applied to model frequency data for single responses (e.g., lever pressing), which will require only minor adjustments in the periphery of the model, which might be a promising endeavor for the future development of the PEP and also for transferring the law of recency to the large literature that uses response frequency as the main dependent variable.

#### Habits Based on Repetition vs. Reinforcement

Given that CL in our experiments was obtained without linking responses to rewards, the resulting behavior reflects an instance of repetition-based rather than reward-based habits (Thorndike, 1898). Establishing habits without linking behaviors to rewards is an interesting finding in and of itself, showing that reinforcement is not a necessary condition for habit acquisition. On the other hand, we cannot say anything definitive on the possible effects that rewards may have (or not have) on episodic response retrieval processes on the basis of our study, since we did not manipulate rewards.

Separating effects of rewards from repetition can be difficult since reinforcement cannot be applied in the total absence of behavior and typically leads to a higher frequency of showing the respective behavior in the situation in which it was rewarded. As soon as frequent repetitions are involved, however, episodic response retrieval may come into play, and may explain the resulting effects. Our experimental paradigm offers an elegant solution to address this problem in future studies: Systematically varying the response that had to be performed during the last occurrence of a stimulus and either rewarding (or punishing) it or not allows for a systematic investigation of the effects of episodic binding/retrieval, reinforcement, and their interaction (preliminary evidence of a recent study, however, suggests that reinforcement of SR combinations does not have a positive effect on the strength of the resulting binding and retrieval effects; Hauber, 2019).

#### What Is a Reward?

Although responses that are based on the contingencies of the task are not reinforced by tangible external rewards (e.g., money), it may still be the case that they are reinforced more indirectly, in that responding in line with the contingencies on average leads to performance benefits (i.e., faster responding). Our findings demonstrated that responding is faster in trials that confirm the contingency in comparison to those trials that are exceptions to the rule. Given that trials confirming the rule are more frequent, this effectively leads to a performance advantage. In our view, however, this difference is not yet evidence for a general performance benefit due to the contingency. The RT difference between high and low frequency trials does not reflect a difference between responses following the contingency rule and those that do not. Instead, both responses follow the contingency rule. The fundamental rationale of the CL paradigm is that contingencies affect responding in all trials, since participants do not know which sort of trial will be presented next. If participants were not influenced by the contingencies also in the low frequency trials, there probably would have been no difference because there were no costs. The claim that participants profit (overall) from applying the contingency rule requires a comparison with a no contingency condition, which was not part of our design. We thus can only speculate on whether it is plausible to assume that behavior in line with the contingency rule is rewarded. In our view, this is unlikely for our study, for the following reasons. First, due to the weak contingencies that we applied in our study, the difference in RTs between high and low frequency trials (which is the upper limit for a performance benefit in comparison to a no contingency baseline) were very small (less than 10 ms), and might not even be perceptible for participants. Second, although there may be a (negligible) performance benefit with regard to speed, responding in line with the contingencies also comes with a somewhat less negligible cost regarding errors. In Experiment 2, absolute error rates were 1.6% higher in the low frequency trials, which is a 20% increase given that overall, about 8% errors were made. Of course, we cannot exclude with certainty that the contingency might also have a beneficial effect on accuracy in the high frequency trials, but such an effect is somewhat unlikely, because the contingency favored not just one but two responses, which should increase error percentages even in high contingency trials.

We also investigated whether the contingency manipulation has an effect on the immediate trial-by-trial feedback that participants received during the task, and whether this might have affected the resulting CL effects. Participants received feedback (a) when their response was slow (i.e., above 1,000 ms) or (b) when they responded incorrectly. With regard to the speed-related feedback (i.e., “too slow” messages), the contingency conditions did not differ significantly in either of the two experiments, due to the fact that the CL effect was small in absolute size and responses were faster than the response deadline in most of the cases for both high and low frequency trials. For error feedback, there was no difference in errors between the contingency conditions in Experiment 1 (and thus no difference in error-related feedback either), but there was a small but significant effect (1.6%) in errors between the high and low frequency conditions (corresponding to a difference in error-related feedback) in Experiment 2. Controlling for this difference at the subject level, however, did not alter the CL effect for RTs at all, nor did it change any of the results of the other analyses regarding the effects of the previous occurrence. Most importantly, the interaction between CL (at the trial level) and the error-related feedback effect of the contingency manipulation (at the person level) did not interact (*t* < 1), indicating that the CL effect was completely independent of the difference in error-related feedback that participants received. Apparently, the CL effect is unrelated to any feedback participants received.

Assuming for a moment that contingencies may nevertheless come with overall performance benefits (compared to a no contingency baseline that was not part of our study), since they reduce uncertainty, then what can one do about it? A straightforward solution would be to eliminate (extinction) or even reverse (countercondition) the contingency for some time, similar to an outcome devaluation procedure in a study of operant conditioning (cf. Schmidt et al., submitted). Based on our findings, however, this is probably not a promising strategy, since episodic retrieval is influenced by responses that were given during the last occurrence of the situation (“law of recency”). Eliminating or reversing the contingency should thus eliminate or reverse the direction of retrieval processes, effectively destroying the effect. Another somewhat speculative possibility might be to incentivize speed or accuracy in different parts of the experiment, but to keep the contingencies constant. Assuming that contingencies produce mostly gains in speed but mostly costs in accuracy, this should effectively reverse the reinforcement logic, but will keep the basic S-R contingencies intact.

#### The Question of Automaticity

A crucial question regards the implicitness or non-intentional (i.e., non-instrumental) nature of CL, since this is a precondition of considering it as an instance of habit formation. As we have explained in the introduction, habits reflect stimulus-driven operant behaviors that are characterized by features of automaticity. Research on habit acquisition often relies on using outcome devaluation as a crucial test for establishing the habitual character of a behavior (e.g., Moors et al., 2017; De Houwer et al., 2018). This criterion is of utmost importance when behaviors have previously been reinforced or are still followed by certain outcomes. Without establishing persistence and stability of the behavior in question independently of the rewards (i.e., after outcome devaluation), a core criterion of automaticity cannot be claimed, which is goal-independence. The resulting behavior may thus still have an instrumental character, which speaks against its purely habitual character. In our view, however, outcome devaluation is not a necessary criterion of habit acquisition. Only when behaviors are or have been linked to rewards can the criterion of outcome devaluation be directly applied. If habitual (i.e., automatic) operant behavior can be established via learning or experience without involving reinforcement (as we would argue is the case for the current study of CL without tangible rewards), then the test of outcome devaluation is not directly applicable (if there is no reward, then it cannot be devalued). Although tests of outcome (in-)dependence can be *added* to investigate the reward sensitivity (goal dependence) of a behavior, such a test cannot question the reward independence of the original behavior, which has been established in the absence of rewards. Demonstrating an influence of reinforcement does not explain why habitual responding was found in the absence of rewards in the first place. This becomes immediately evident when considering outcome devaluation procedures, where outcome devaluation typically does have a strong effect on responding – the crucial aspect is that it does not eliminate behavior *completely*.

However, if the question of goal-independence and the test of outcome devaluation do not directly apply to our study, because contingencies were not rewarded in the first place, what is the basis on which we claim that CL results in a habit, that is, is automatic? CL has been shown to operate in the absence of awareness, which is a major criterion for automaticity (Schmidt et al., 2007). In the current studies, we used weak contingencies, which should be much harder to detect than the contingencies that were used in the study by Schmidt et al. (2007). In addition, we made the contingencies more complex, by making each word predictive of two instead of only one color, which should prevent participants from translating the contingencies into simple response strategies (cf. Schmidt and De Houwer, 2016). Finally, our study capitalized on yet another criterion of automaticity, which is speed. By introducing a response deadline of 1,000 ms, we exerted time pressure on participants during the task, which limits controlled processes during the task to a minimum, and has been shown to foster habitual responding (Hardwick et al., 2018; Luque et al., 2019). In sum, we thus feel justified in claiming that the CL effects that were obtained in our study reflect the operation of automatic processes, and thus can be characterized as being implicit. Of course, we have to acknowledge the limitation that we did not include any direct measures in our study that allowed us to conduct an empirical test for one or more criteria of automaticity within our experiments (Moors and De Houwer, 2006).

To sum up, we want to emphasize that our study is based on a broad conception of habits that categorizes operant behavior as habitual if it is stimulus-bound and shares some features of automaticity. This usage differs from a more narrow conception of habits that has been proposed by some researchers in the field (most notably, Dickinson, 1985), who argued that goal-independence is the core criterion of a habit, and that outcome devaluation is a necessary test to establish the habitual character of a behavior. It is important to interpret our findings against this background. Since we employed different criteria of habitual behavior, our core finding that habits can be explained in terms of episodic response retrieval may not generalize to habits that were established in terms of outcome independence. Further research is needed to clarify whether this functional explanation can be transferred also to behavior that has been shown to be goal-independent.

#### Stimulus Dependence, and Relevance of the Situational Cues

On a more general level, our results also bear some important implications for our understanding of habit formation. In particular, our findings highlight that situational cues play a crucial part in the acquisition and maintenance of habits, even when these situational cues are completely irrelevant for the performed behavior. This is supported by the fact that word meaning was irrelevant for the color categorization task in the present study. However, participants’ performance showed that they were sensitive to the co-occurrence of words and responses, and automatically retrieve the episodic instance in which the current word was presented most recently. Our findings reveal that effects of CL do not imply that participants were making strategic use of these regularities (cf. Schmidt et al., 2007; Giesen and Rothermund, 2015; see our arguments above). Apparently, all it takes to produce these effects is retrieval of the last occurrence of the word from episodic memory in order to simulate global CL effects (Schmidt et al., submitted).

#### Moderating Effects of Distance

Our study provides support for the law of recency by demonstrating that habits can emerge on the basis of retrieving just one single episode, which is one in which the person has responded to the current stimulus when it had been encountered during its last occurrence. In a situation where the last encounter has been fairly recent, this effect is strong enough to override all other previous occurrences of this situation that occurred before the last occurrence, rendering global contingencies irrelevant. However, as our data show, the last occurrence of a stimulus/situation quickly loses its influences on behavior with increasing distance to the current situation. Our findings revealed that after 5–6 intervening trials the influence of the last occurrence already vanishes. It remains unclear what happens if the last occurrence exceeds this distance: Instance-based retrieval might either break down completely for long intervals; alternatively, retrieval might still operate but might no longer be restricted to the very last occurrence (cf. Schmidt et al., 2016). According to the latter alternative, the last episode becomes less distinct with increasing distance and will more easily be confused with other instances. The predictions of these two alternatives are starkly different: According to the first variant, global contingencies will not influence behavior at all after controlling for the last occurrence, whereas the second account would predict that effects of mere frequency and/or global contingencies become visible when the last occurrences of the situation is distant. In this case, contingency effects would still reflect retrieval, but retrieval becomes less selective and will resemble more and more the probabilities and contingencies that are inherent in the entire set of previous episodes that share features with the current situation.

#### Relation Between the Laws of Recency, Exercise, and Effect

Our findings should not be taken to indicate that large frequencies of executing the same behavior over and over again (“law of exercise”) have no influence on habitual behavior. For one thing, we did not test any influence of massive repetitions in our studies. We do not have any evidence on this, but it might well be that repeating a response for, say, more than 500 times might result in such a strong habit that inserting one counter-example might not suffice to overcome it. In fact, the influence of massive repetitions might be mediated by a different pathway, and might operate independently from episodic retrieval process. On the other hand, instance-based retrieval processes and the law of recency might also play an important role for the explanation of overlearned behaviors. To test such an assumption, experiments should vary the similarity between the contexts in which the behavior was repeated and when it is tested. If exercise-based habits are shown to be context-dependent, then retrieval processes might also play a role in explaining these effects, but as we said, that remains to be investigated in future studies.

Finally, we also want to highlight that our findings do not rule out that instrumental behavior is influenced by rewards and incentives (“law of effect”). Demonstrating habitual behavior in the absence of reward just shows that reinforcement is not a necessary ingredient of habitual behavior (similar to what previous research has already shown with the outcome devaluation test). It could well be that reinforcement has a strong influence on responding also in the CL task, and it could also be that processes of episodic retrieval and CL are influenced by systematically rewarding or punishing certain combinations of stimuli and responses (but see Hauber, 2019). Demonstrating habitual behavior in the absence of rewards, however, attests to the fact that reinforcement is not a necessary ingredient of habits.

### Practical Implications

The present findings also have important practical implications for the emergence and change of habitual responding. As shown in the present experiments, (irrelevant) situational cues play a major role in the acquisition and maintenance of habits. With regard to practical implications, this insight renders “exposure management” or “situation control” as another key variable of habit change. This claim is supported by research showing that a change of context reduces habitual responding in rats (Thrailkill and Bouton, 2015) and also in humans (e.g., Wood et al., 2005; Verplanken et al., 2008). Interestingly, gaining control over situational retrieval cues (e.g., creating a “seating habit” of sitting with one’s back to an all-you-can-eat buffet; Wansink and Payne, 2008) has the potential to become a new, desirable habit that counteracts undesirable habits (like unhealthy eating) in the future.

The core finding of our study is that the most *recent* stimulus-response bindings are crucial for the maintenance of habitual behavior, attesting to the law of recency. This reasoning is supported by the finding of the current study, as well as others (Schmidt et al., submitted), that response retrieval is much stronger for short distances, and that CL effects seem heavily influenced by more recent bindings. Put differently, in our study it was not the *frequency* of a pairing (reflecting global SR contingencies) but the *recency* of the episode that determines the direction of the habitual impulse. Our findings thus attest to the enormous importance of the very last occurrence of a certain situation in determining the response that is retrieved. Each word stimulus occurred hundreds of times during each experiment, and was paired with four different responses, two of which were highly frequent. Still, response retrieval was driven more or less completely by the last occurrence of the word, and focusing on only the last occurrence was sufficient to fully explain CL, that is, habitual responding.

The strong effects of recency and in particular the behavior that was shown during the last occurrence of a certain situation offers important insights that can be inspiring for interventions targeted at creating desirable or breaking undesirable habits (for an overview, see Wood and Rünger, 2016): Executing a new behavior only once should already have a strong effect on subsequent behavior in this same situation. This strong effect is well-known for piano players who often have the (deplorable) experience that a specific error which occurred for the first time (and only once) at a certain point in a piece of music then has an extremely strong tendency to repeat at the next time, and to become chronic (see Marx, 1971; Marx et al., 1973; Marx and Marx, 1980; for a review, see Koppenaal, 1960).

On the other hand, this strong effect of a single episodic occurrence also offers a chance to change a bad habit into a good one by changing behavior only once. Breaking or overcoming existing habits typically requires effort and concentration (executive control). Our findings support the view, however, that spontaneous retrieval kicks in after only one occurrence and that execution of a response in a situation then impacts later behavior when the situation is encountered again.

At the same time, however, it would probably be naïve to assume that a strong habit is already formed just by changing behavior once, and then trusting in retrieval of the last occurrence. Although we would assume that such a strategy may work remarkably well for the context in which the behavior is changed for the first time, it may not work anymore once the behavior has been interrupted by some other activity. It is not that episodic retrieval would not operate across large temporal distances (see previous section). Quite the contrary, the fact that habits are so robust already shows that time alone does not interfere with retrieval. What is different with increasing time is that the advantage of the last response episode – compared to the other episodes that were stored in memory before the last episode – is eliminated. The sharp decay function of episodic retrieval yields a clear advantage for the last episode across short time intervals; across longer intervals, however, the overall contingency should determine retrieval probabilities. That is, changing a habit once will typically be followed by immediate marked changes in behavior. To change it in the long run, however, will require repeated attempts in each new situation until the overall contingency has switched toward the new behavior.

## Data Availability Statement

The datasets generated for this study are available on request to the corresponding author.

## Ethics Statement

In accordance with ethical standards at the FSU Jena, the study was exempt from further ethical approval because no cover-story or otherwise misleading or suggestive information was conveyed to participants.

## Author Contributions

CG co-developed the research idea, study, design, organized the data collection and analyses, and prepared the manuscript. JS involved in manuscript preparation. KR co-developed the research idea, study, design, involved in data analyses and manuscript preparation.

## Conflict of Interest

The authors declare that the research was conducted in the absence of any commercial or financial relationships that could be construed as a potential conflict of interest.

## Footnotes

1 Researchers in the tradition of the law of exercise have claimed that outcome devaluation (Adams, 1982) is a necessary criterion for establishing that behavior is habitual (e.g., Dickinson, 1985; Ostlund and Balleine, 2007). Although we agree that demonstrating the stability of a behavior against outcome devaluation is important for establishing that a certain behavior is habitual rather than instrumental, we do not think that it should be considered to be a necessary criterion to establish behaviors as habitual. In some cases (e.g., the present study), behaviors are not systematically linked to any (positive or negative) outcomes in the first place. If habitual behavior is established in the absence of reinforcement, the outcome devaluation procedure is not directly applicable (if there is no reward that is linked to the habitual behavior in question, then it cannot be devalued). In addition, alternative criteria can be used to establish that the behavior in question shares features of automaticity (e.g., unawareness, efficiency [resource independence]), and thus can be established as being habitual.

2 A similar rationale is used in studies investigating the competing influence of local and global contexts on thought and action (e.g., Meier and Kane, 2013; MacLellan et al., 2015; Fröber et al., 2018).

3 Another reason for making each word predictive of two colors was to investigate stimulus-response binding and retrieval effects for immediate sequences in a design that is maximally balanced with regard to contingency as well as stimulus- and response relations. This aspect of our studies, however, was not the core focus of the current paper.

4 Probe RT below 250 ms or more than 1.5 interquartile ranges above the third quartile of the individual RT distribution were regarded as outliers (Tukey, 1977). Note that results were virtually identical when trials were filtered according to the “far out” criterion (i.e., exclusion of RTs more than 3 interquartile ranges above the third quartile of the individual RT distribution) or when sample based RT distributions were used instead of individual RT distributions.

5 https://aspredicted.org/blind2.php

6 This main effect supposedly reflects a combination of two things: (a) response retrieval effects due to the repetition of a word are stronger for short (i.e., immediate repetitions) than for larger distances, and (b) benefits of retrieving a correct response outweigh the costs that are incurred due to retrieval of a different response (for a discussion, see Giesen and Rothermund, 2016). In sum, this leads to a facilitating effect of word repetitions on performance that is stronger for immediate than for distant repetitions.

7 A possible reason for these additional interactions may be that the asymmetry of benefits and costs that are due to retrieving the correct response are stronger for high than for low contingency trials. The correct responses that are retrieved in the high contingency condition are responses that have often been paired with this word, whereas the correct responses that are retrieved in the low contingency condition are responses that have been paired with this word only rarely. Conversely, the wrong responses that are retrieved in the low contingency condition are mostly those responses that have frequently been paired with this word before.
